# Strengthening Community-Based Vital Events Reporting for Real-Time Monitoring of Under-Five Mortality: Lessons Learned from the Balaka and Salima Districts in Malawi

**DOI:** 10.1371/journal.pone.0138406

**Published:** 2016-01-11

**Authors:** Olga Joos, Agbessi Amouzou, Romesh Silva, Benjamin Banda, Lois Park, Jennifer Bryce, Mercy Kanyuka, RMM Malawi working group

**Affiliations:** 1 Institute for International Programs, Department of International Health, Johns Hopkins Bloomberg School of Public Health, Baltimore, Maryland, United States of America; 2 Division of Policy and Practice, UNICEF, New York, New York, United States of America; 3 World Food Program, Lilongwe, Malawi; 4 Malawi National Statistical Office, Zomba, Malawi; Vanderbilt University, UNITED STATES

## Abstract

**Background:**

Malawi ratified a compulsory birth and death registration system in 2009. Until it captures complete coverage of vital events, Malawi relies on other data sources to calculate mortality estimates. We tested a community-based method to estimate annual under-five mortality rates (U5MR) through the Real-Time Monitoring of Under-Five Mortality (RMM) project in Malawi. We implemented RMM in two phases, and conducted an independent evaluation of phase one after 21 months of implementation. We present results of the phase two validation that covers the full project time span, and compare the results to those of the phase one validation.

**Methods and Findings:**

We assessed the completeness of the counts of births and deaths and the accuracy of disaggregated U5MR from the community-based method against a retrospective full pregnancy history for rolling twelve-month periods after the independent evaluation. We used full pregnancy histories collected through household interviews carried out between November 2013 and January 2014 as the validation data source. Health Surveillance Agents (HSAs) across the 160 catchment areas submitted routine reports on pregnancies, births, and deaths consistently. However, for the 15-month implementation period post-evaluation, average completeness of birth event reporting was 76%, whereas average completeness of death event reporting was 67% relative to that expected from a comparable pregnancy history. HSAs underestimated the U5MR by an average of 21% relative to that estimated from a comparable pregnancy history.

**Conclusions:**

On a medium scale, the community-based RMM method in Malawi produced substantial underestimates of annualized U5MR relative to those obtained from a full pregnancy history, despite the additional incentives and quality-control activities. We were not able to achieve an optimum level of incentive and support to make the system work while ensuring sustainability. Lessons learned from the implementation of RMM can inform programs supporting community-based interventions through HSAs in Malawi.

## Introduction

The Millennium Development Goals have catalyzed attention and action to address key health and development issues, while improving the evidence base for holding countries accountable for development progress by tracking results. Attention to maternal and child health commitments has continued, with new pledges to maintain engagement in reaching maternal and child survival goals [1,2]. High-quality and timely data are needed to track progress toward these goals. The ideal source of mortality data is comprehensive vital registration systems with at least 90% coverage of births and deaths [3]. However, only 30% of the world’s population lives in countries with complete birth and death registration [3]. Calls for strengthening vital registration processes have been made simultaneously with maternal and child health commitments, but have only recently been presented with investment and scale up strategies for guidance [4]. The creation or strengthening of a vital registration system is not an easy feat given complex health system weaknesses, bureaucratic inertia, and the range of health issues vying for program support and funding. Moreover, some socio-cultural beliefs at the community level act as barriers to registration [5–8].

In Malawi, efforts began in 1983 to change the 1904 Births and Deaths Registration Act, requiring only the registration of non-Africans, and making registration of Africans voluntary [9]. In 2009, Malawi ratified a compulsory and free birth and death registration act which incorporates health system activities and support into birth registration. Parents must submit the birth report they receive at birth or during a postnatal check-up to the district registrar within six weeks of birth for free registration and to avoid a fine [10]. Considerable work remains to fully implement this system and improve the completeness and quality of birth and death registration data [11]. In the meantime, Malawi is using other demographic and health data sources to inform policy and program decisions. The Malawi National Statistical Office (NSO) relies on the population census and household surveys such as the Demographic and Health Survey and Multiple Indicator Cluster Survey to estimate population and health statistics but these high-quality surveys with national and district level results are only conducted every three to five years [11]. Modeling is used in interim periods but does not generate subnational estimates. A Health and Demographic Surveillance Site was established in 2002 in the Karonga district of northern Malawi that provides complete and high-quality vital events surveillance of its catchment population, but was not designed to produce estimates representative at the national level [12].

An ideal interim mortality data source should generate national and district level mortality estimates with cause of death [13]. With an estimated 11,000 Health Surveillance Assistants (HSAs) located in every catchment area throughout the country, Malawi has a workforce within the health system to capture births and deaths. In fact, HSAs are a *de facto* vital event documentation system. Their scope of work includes tracking pregnancies and documenting births and deaths in their Ministry of Health-issued Village Health Register, referred to as register in this publication [14]. HSAs have the potential to be a source of district-level vital events data, but there has not yet been any systematic evaluation of the timeliness, completeness, and accuracy of vital events data reported by HSAs.

From January 2010 to December 2013, the NSO and the Institute for International Programs at Johns Hopkins University implemented the Real-Time Monitoring of Under-Five Mortality (RMM) project among a random sample of HSAs in the Balaka and Salima districts of Malawi. These districts were selected due to their high fertility, high under-five mortality, HSA placement in all catchment areas, average district population size, and easy access for the study team based at the NSO in the Southern Region. Details on the sampling procedures are described in detail elsewhere [15]. The main objective of RMM was to assess the accuracy and reliability of HSA routine reports as a source of data on district-level under-five mortality in a high mortality and fertility setting. If successful, the government planned to expand the approach to other districts in Malawi as an interim source for district-level estimates of under-five mortality rates (U5MR), an important indicator reflecting socio-economic development. As a collaborator in the project, the Ministry of Health was interested in assessing whether HSAs were able to perform the part of their job description that requires reporting on vital events to potentially support the strengthening of the civil registration and vital statistics system.

A first phase of RMM covered the period of time between 2010 and 2012, and validation analyses were conducted and published elsewhere [15]. A second phase with enhanced support was implemented between September 2012 and December 2013. The current research is an extension of the earlier phase one validation analysis and covers the full phase one and phase two periods. Here we describe the context, methods, results, and lessons learned from the community-based RMM method implemented from January 2010 through December 2013 in the two districts.

## Methods

### Program Design

Malawi is a landlocked country in east-central Africa with an estimated population of 13 million inhabitants, 85% of whom live in rural areas [16]. Administratively, Malawi is divided into 28 districts, which are further divided into traditional authorities ruled by chiefs. Health care is provided at district health centers and central hospitals with primary outreach care provided by HSAs, of which 50% should be female. HSAs are required to have a minimum of ten years of education and to complete a 12-week training course, during which they receive a register issued by the Ministry of Health in which to document their work [17]. HSAs are responsible for health promotion, treatment, and health surveillance in catchment areas that cover approximately 1,000 inhabitants [18]. They are expected to reside in their catchment area and are linked to a nearby health facility. Table 1 presents HSA and catchment area characteristics collected in two cross-sectional HSA surveys conducted in the final year of the project.

**Table 1:** HSA and catchment area characteristics.

	Balaka*	Salima*	Total*
**Health facilities**			
Number of health facilities	13/30 (44%)	17/30 (57%)	30
HSAs working on RMM per HF	5 (±4), 1–14	4 (±6), 2–19	5 (±4), 1–19
(mean ± SD), range (HSAs)			
**HSA characteristics**			
Sex			
Female	24/80 (30%)	28/79 (35%)	52/159 (33%)
Residence			
HSA residence in catchment area	33/79 (42%)	39/78 (50%)	72/157 (46%)
**Catchment area**			
Turnover during project period			
Catchment areas with HSA turnover	17/80 (21%)	27/79 (34%)	44/159 (28%)
HSAs with supervisor turnover	56/79 (71%)	46/76 (61%)	102/155 (66%)
HH population	(*N* = 79)	(*N* = 79)	(*N* = 158)
Estimated population in HSA catchment	1455 (±443),	1465 (±606),	1458 (±535),
area (mean ± SD), range (inhabitants)	485–2600	635–4030	485–4030

*Unless otherwise specified, values are numbers (%).

HSAs track pregnancies, births, and deaths in their community through active surveillance conducted as household visits and regular meetings with Village Health Committees in their catchment area. They also have many other tasks, including health education, disease surveillance, prevention and treatment of specific childhood diseases, and monitoring of the water supply and community sanitation, that demand varying amounts of HSA work time [14,19]. The RMM community-based approach relies on HSAs’ active surveillance of births and deaths in their register to calculate yearly estimates of under-five mortality.

**Fig 1:**
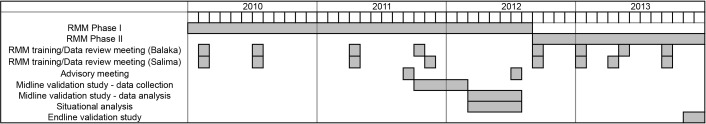
RMM Timeline. Timeline of RMM implementation, validations, and meetings from January 2010 through December 2013.

The RMM method was developed and implemented in two phases in two districts, Balaka and Salima. Phase one covered the period from January 2010 through August 2012, and included a midline validation survey with a full birth history conducted between October 2011 and February 2012. Details of the initial formative research, study design, phase one implementation, and midline validation survey results are presented elsewhere [20]. Due to underreporting of births and under-five deaths revealed in the midline validation, a situation analysis was conducted in collaboration with the Ministry of Health to identify adjustments needed to the approach for increased reporting and completeness in the mortality results (S1 File). Results from phase one and the situation analysis informed the implementation of phase two, with an enhanced strategy and strengthened incentive structure for HSAs and supervisors. Phase two ran from September 2012 to December 2013. An endline validation survey with a full pregnancy history was conducted between November 2013 and January 2014 to validate data from the complete project timeframe and to provide a basis for comparing results from phases one and two. Fig 1 shows the timeline for project activities in Malawi.

### Stakeholder Engagement

Stakeholder engagement was prioritized throughout the course of the project to ensure the most effective design and implementation of the community-based RMM method. An advisory group consisting of Ministry of Health, NSO, UNICEF, and Institute for International Programs representatives was established to inform and oversee the development and implementation of the project. The advisory group met at pivotal points throughout the course of the project, and used reports and results from RMM activities to guide their recommendations. Additionally, some stakeholders in the advisory group participated in the data review meetings, which broadened their understanding of the project beyond reports, and incorporated field work successes and challenges discussed by HSAs, supervisors, and district coordinators. These stakeholders used their comprehensive understanding of the HSAs’ role within the Malawi health system, the project, and its results to redesign RMM between phase one and phase two, and to identify key conclusions of the project which were shared with a broader audience at a dissemination meeting in November 2014.

### Data Collection Process

**Fig 2:**
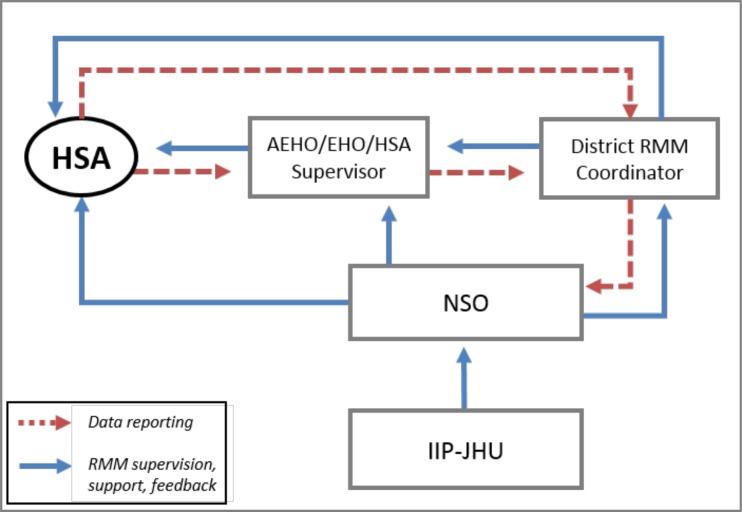
Organizational chart. Organizational chart of RMM data flow and supervision among HSAs, Assistant Environmental Health Officers (AEHO), Environmental Health Officers (EHO), supervisors, district RMM coordinators, and team members at the NSO and the Institute for International Programs at Johns Hopkins University.

Within the scope of work set by the Ministry of Health, HSAs are expected to identify pregnancies, births, and deaths within their catchment areas during monthly visits to households and Village Health Committees in their catchment area. Through this active surveillance they identify events and record the information in their register. RMM reinforces these tasks and adds only an additional step of extracting pregnancies, births, and deaths each month from the register onto a monthly extraction form. Fig 2 shows the routine flow of information and monitoring/feedback processes associated with RMM. Details of the data management process and supervision standards are discussed in S2 File. Double data entry was conducted in CSPro and was followed by reconciliation conducted by the data editor and cleaning conducted by the NSO principal investigator [21].

### Supervision

Supervision was an important component of the data quality-control process and planned as a frequent supportive activity. The district RMM coordinator assigned each HSA working on RMM to a specific supervisor at the health center and identified a replacement supervisor if a supervisor left. Supervisors participated in the initial RMM training in February 2010 and subsequent data review meetings.

Supervisors were responsible for visiting the HSAs in their catchment areas each month to check their extraction forms along with the registers, remind HSAs of data quality guidelines, and collect the extraction forms for submission to the district RMM coordinator. Supervisors did not receive supervision from district RMM coordinators, but did receive additional support in phase two with a supervision checklist, which standardized the supervision visit and improved tracking of supervision. Supervisors who performed well as measured by the completion and submission of supervision checklists were recognized at the data review meetings as “Supervisors of the Quarter” and were given additional cell phone credit as a reward.

### Training

Data review meetings were held bi-annually throughout phase one in both districts to review data and re-train HSAs and their supervisors on data quality guidelines. All HSAs and supervisors were invited to the review meetings, along with Ministry of Health officials, the District Health Officer, the Health Management & Information Systems officer, and other partners such as UNICEF and WHO. During the review meetings, Ministry of Health representatives and NSO data management team members reviewed the HSA scope of work, project results by district since the start of RMM, and data quality guidelines to reinforce the importance of ensuring data quality in terms of accuracy, reliability, and completeness.

The findings of the situation analysis indicated that the data review meetings were an important incentive for both HSAs and their supervisors. In phase two, which was formally introduced at the September 2012 data review meetings, we increased the frequency of these meetings and they were planned for every quarter. Strengthening community ties and the data management process became a priority in phase two. The RMM data management team at the NSO led the trainings of the Village Health Committee to orient them to RMM and to promote collaboration with HSAs. We implemented an mHealth intervention to improve data quality with a focus on improving the documentation of pregnancy outcomes. Phase two incentives, activities, and training details are described in Table 2.

**Table 2:** Description of phase two improvements and rationale.

Phase II incentive category	Activity or material	Reason
Supplies	Carbon copy extraction form booklets	To improve efficiency in submission of replacement form if original form misplaced
	Village health registers	To meet HSA need for new VHRs
	New backpacks	To promote the carrying of VHRs during household visits
Supervision Support	Quarterly "Supervisor of the Quarter" award	To incentivize regular supervision
	Supervisor checklist	To provide guidance and structure to supervision visits
HSA feedback and incentives	Quarterly HSA report cards	To provide feedback on documentation accuracy, timeliness, and completeness
	Quarterly "HSA of the Quarter" award	To incentivize complete and accurate documentation
	Regular data quality SMS	To remind HSAs of RMM work and good documentation practices
Community engagement	Village Health Committee (VHC) training on RMM	To improve collaboration in sharing vital events data mong HSAs and VHCs

A final data review meeting was held in June 2014, during which complete project and validation results were presented and discussed. Participants also provided recommendations for improvement if RMM is continued or scaled-up. The advisory group reviewed this feedback at its final meeting in June 2014 to incorporate with key findings at the dissemination meeting in November 2014.

### Village Health Register Verification Surveys

We conducted two field-based assessments of HSA data recording practices to evaluate the accuracy of HSAs’ use of their register to record events and extract them onto the RMM forms. The first assessment was conducted during phase one, in January 2012, and the second during phase two, in August 2013. All 160 RMM HSAs were included in the assessments. In each assessment, we checked all births reported by HSAs to the NSO for RMM in the past three months and under-five deaths reported in the past 12 months against the events recorded in each HSA's register. We then confirmed all under-five deaths and up to three randomly selected births by visiting the household where they occurred. Details of survey methods and results are available in S3 File.

### Validation Surveys

Two household surveys were conducted during the course of the community-based RMM project to assess the validity of the events reported by the HSAs. We describe the surveys briefly here; details are available in S4 File. The midline survey included a full birth history and was conducted from October 2011 to February 2012. We needed a sample size of 30,000 households (increased by 7% to account for transfers and loss to follow-up) to validate community-based RMM using the midline survey results. Trained interviewers conducted the survey using paper questionnaires, with questions in English and Chichewa.

The endline survey included a full pregnancy history and was conducted between November 2013 and January 2014. We needed a sample size of 10,000 women for the pregnancy history. The survey was conducted using computer-assisted personal interviewing technology. Interviewers were trained in administering the questionnaires and in basic computer skills. The survey questions were in English and Chichewa, although most interviews were conducted in Chichewa.

We assessed the validity of the HSA data by estimating under-five, infant, and neonatal mortality rates from the HSA data for rolling 12-month periods, and comparing the results with those estimated from each validation survey. A maximum difference of 20% between the U5MR generated by the RMM method and the validation surveys was required to consider RMM successful. We also examined the completeness of HSA reporting for births and under-five deaths by analyzing the ratio of the total number of births and under-five deaths documented by HSAs to the expected number estimated from each validation survey. We assessed the quality of the births and deaths data collected by HSAs by comparing core indicators, such as the sex ratio at birth and age distribution at death, with those estimated from the validation surveys.

### Ethical Review

Ethical approval was obtained in the U.S. by the Institutional Review Board at the Johns Hopkins University Bloomberg School of Public Health, and in Malawi by the National Health Sciences Research Committee. For both validation surveys, trained interviewers obtained oral informed consent from each participant in the local language, Chichewa. The Johns Hopkins University Bloomberg School of Public Health Institutional Review Board waived the written consent requirement due to the low literacy of the study population.

## Results

### Validation Surveys

The level of submission of monthly extraction data by HSAs was high. For most months, more than 70 HSAs per district (87.5%) consistently provided extracted data on pregnancies, births, and deaths to the NSO (S2 File).

**Fig 3:**
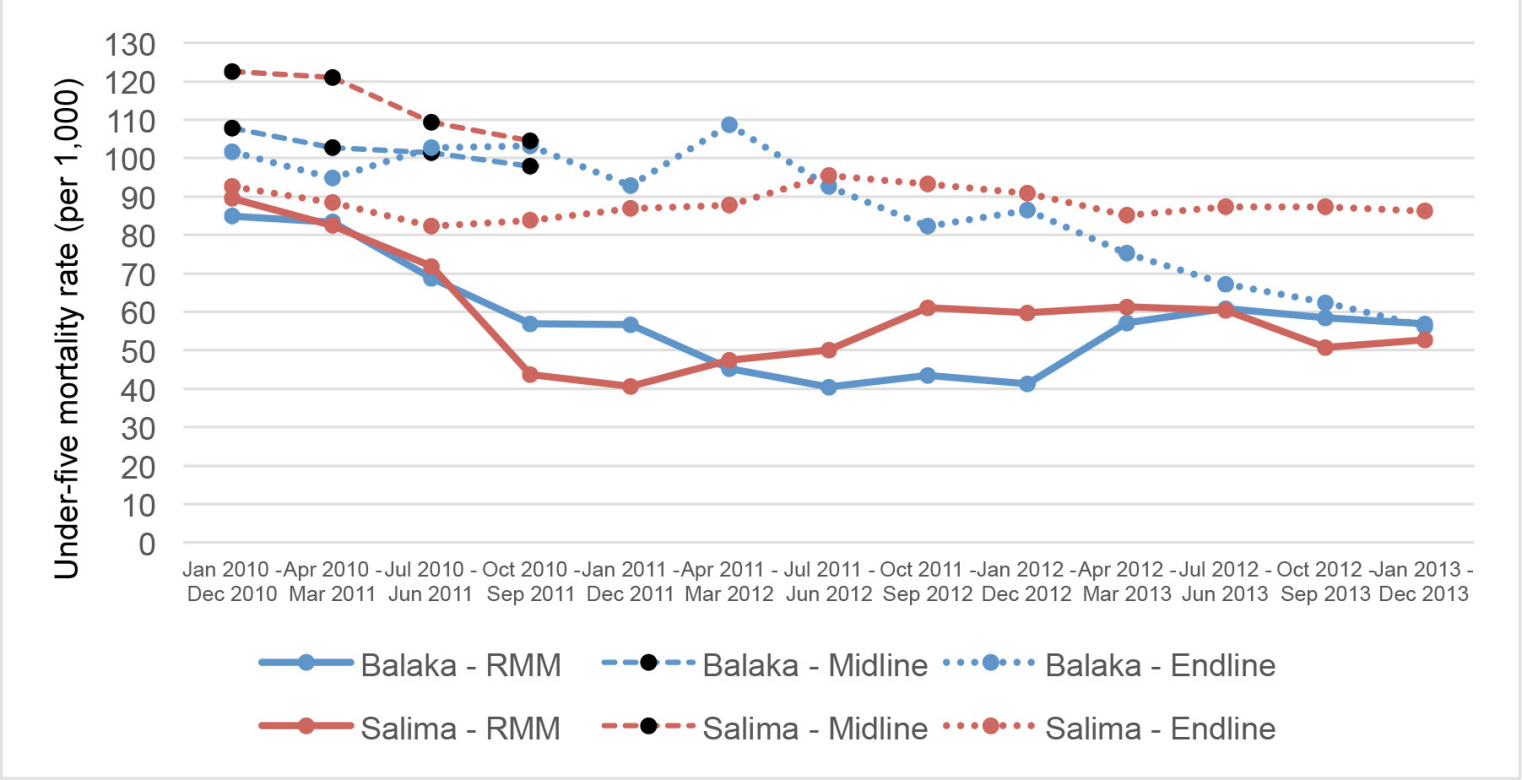
Under-five mortality estimates. Under-five mortality rate estimates, RMM and midline and endline survey data, for annual periods from January 2010 through December 2013 (per 1,000).

Fig 3 compares the U5MR estimates based on HSA data with those estimated using the midline and endline validation surveys. There are some notable differences between the U5MR validation results for Balaka and Salima. The HSA-based U5MR estimates for the first two validation periods (January to December 2010 and April 2010 to March 2011) are within 20% of both the associated endline and midline survey estimates. However, for the subsequent validation periods up to and including January 2012 to December 2012, the HSA-based estimates systematically underestimate U5MR compared to those calculated using the endline validation survey. For the last four validation periods, the U5MR estimates based on HSA data appear to be more consistent with those estimated from the endline survey. However, the last four estimates from the endline survey suggest a consistent decline in U5MR by approximately 45% within 15 months that looks implausible. In Balaka, aside from the first validation periods, the HSA data systematically underestimated U5MR relative to the endline survey.

In Salima, the HSA data, on average, systematically underestimated U5MR relative to the endline validation survey. Also in Salima, in contrast to Balaka, there are inconsistent validation results for the periods for which the endline and midline survey reference periods overlapped. Validation against the midline survey for the first four annual periods (i.e., January 2010 to December 2010 through October 2010 to September 2011) suggests that the HSA data considerably underestimated U5MR for these four annual periods. Validation against the endline survey suggests that the HSA data estimated U5MR within 10% of those estimated by the endline survey for the first three annual validation periods. Given that these endline survey estimates rely on a longer recall period than the midline survey, and thus may be affected by recall lapse (i.e., underreporting) of child deaths by survey respondents, we consider the midline validation results more accurate. The preliminary results of the combined midline and endline survey validation for Salima suggest that the HSA data systematically underestimated U5MR by 25–50%.

**Fig 4:**
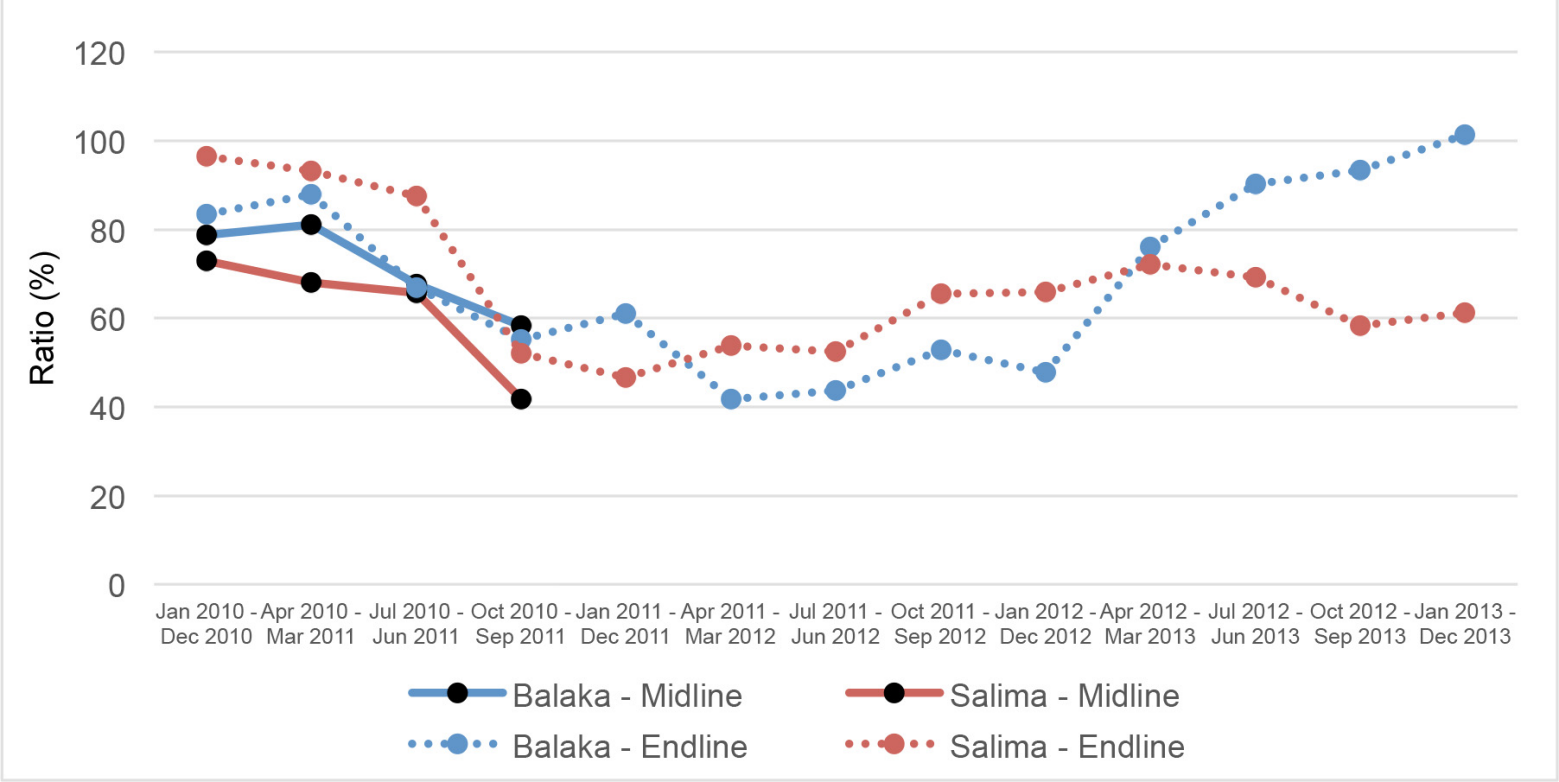
Ratio of under-five mortality rates. Ratio of under-five mortality rates, RMM data to midline and endline surveys, for annual periods from January 2010 through December 2013 (%).

Fig 4 shows the ratio of U5MR generated from the HSA data relative to those generated from the midline and endline surveys. For the last three 12-month annualized periods of the study period, the ratio of HSA-based U5MR estimates to those based on the endline validation survey exceeded 80%. However, this apparent improvement was driven by an implausibly large and rapid decline in U5MR estimated by the endline validation survey in Balaka. Hence, despite some initial promise, the HSA-based vital events reporting generally systematically underestimated annualized U5MRs in both Balaka and Salima.

### Village Health Register Verification Surveys

We verified the content of HSA vital events reported to the NSO with corresponding entries in their registers, and found high levels of consistency between the two sources both in phase one and in phase two (Table 3). In phase one, 92.3% of births reported to the NSO were found in their registers, but this declined to 83.5% in phase two. Death reporting also declined between verification assessments from 90.5% in phase one to 87% in phase two. The only slight improvement between assessments was in the documentation of the date of birth. The date of birth reported to the NSO matched register documentation in 85.9% of cases in phase one and 89.8% in phase two.

**Table 3:** Comparison of select VHR Verification Survey indicators from phase one, January 2012, and phase two, August 2013.

	District				Percentage Point Difference
Variable	Phase one		Phase two		
	n	%	n	%	
Response Rate for HSA questionnaire	160	81.3%	160	93.8%	12.5%
**BIRTHS**					
Proportion of births in NSO records documented in the VHR	1016	92.3%	1211	83.5%	-8.8%
Proportion of births for which the date of birth was consistent with NSO records	1016	85.9%	1211	89.8%	3.9%
Proportion of births confirmed in the HSA catchment area	335	100.0%	412	97.8%	-2.2%
Proportion of births confirmed in the HSA catchment area with consistent date of birth	335	94.9%	385	93.9%	-1.0%
**DEATHS**					
Proportion of deaths in NSO records included in the VHR	423	90.5%	223	87.0%	-3.5%
Proportion of deaths for which the date of death was consistent with NSO records	N/A	N/A	223	91.5%	
Proportion of deaths confirmed in the HSA catchment area	222	98.6%	194	94.8%	-3.8%
Proportion of deaths confirmed in the HSA catchment area with consistent age at death	222	93.7%	194	89.2%	-4.5%
**Residence in catchment area**					
Proportion of HSAs living in catchment area	130	54.0%	150	46.7%	-7.3%

We were able to confirm more than 90% of births and around 90% of under-five deaths reported by HSAs by visiting the household where the event occurred (Table 3). This suggests that the RMM community-based method can produce accurate data when events are fully documented. Verification results from phase two produced slightly lower consistency than those reported in phase one, with associated differences in reporting consistency between the two phases.

## Discussion

These results present an overview of process, data quality, and outcome indicators associated with implementation of RMM community-based methods in Malawi. We have shown that HSAs are capable of documenting and reporting vital events, but they consistently produce underestimates relative to household surveys with women of reproductive age. HSAs generally documented information on births and deaths with a high level of accuracy when they identified and recorded an event. On average, however, over the course of the entire RMM study in Malawi, HSAs underreported births by 44% and under-five deaths by 49%, resulting in an underestimate of the annualized U5MR of about 41%. The completeness of vital events reporting also varied over time. The range of underestimation of the U5MR by HSAs varied between 11% and 59%. Thus, the community-based RMM method did not accurately capture either the levels of, or trends in, childhood mortality. Even after the introduction of additional performance-based incentives for HSAs and increased supervision of the routine vital events reporting procedures in phase two of the study, HSA-based vital events reporting continued to be substantially incomplete and therefore the accuracy of HSA-based U5MR estimates did not improve.

RMM would be an attractive approach to consider as an interim data source to estimate short-term change in U5MR, if accurate results could be produced. RMM reinforces an existing government structure for community-based monitoring of vital events, and avoids creating separate, parallel structures. Additionally, RMM also reinforces the documentation process and the data quality of vital event documentation set by the Ministry of Health, enhancing its potential for sustainability [22,23]. This potential is further strengthened with the engagement of actors at all levels of the community and health system, especially those at the national and community level. Participation by Ministry of Health representatives in RMM review meetings motivated HSAs and improved the HSAs’ understanding of their scope of work, which is constantly evolving with the inclusion of new tasks from the Ministry of Health and local NGOs [22]. With the evolution of the role of community-based health workers and the competing demands on their time that often result in task shifting, the review of work responsibilities has been shown to improve motivation and satisfaction among community health workers [17,22,25,26]. The Ministry of Health should disseminate an HSA scope of work description annually to promote regular assessment of HSA responsibilities at the ministry level and provide job clarity among HSAs, supervisors, and health facility staff at the community level.

Although the approach of using HSAs for monitoring child mortality appears feasible and attractive, it requires a high level of input to ensure HSAs and their supervisors are well-trained and have support, and that incentives and supervision are provided continuously to maintain high levels of performance. Research has found that these inputs are important for job satisfaction and completion of tasks, but alone do not guarantee motivation and engagement [17,24,25]. We did not foresee a need for intensive training of HSAs, because the documentation of vital events was already part of the job description of the HSAs prior to the implementation of RMM. We learned after phase one that considerable additional investments were needed to strengthen the data management process, yet we did not find improvements in under-five mortality reporting in phase two. Full implementation of phase two incentives and activities was not accomplished until July 2013, which may have contributed to the lack of improvement in reporting during phase two.

Both HSAs and supervisors reported that data review meetings were an important incentive. Plans to hold these meetings quarterly in Phase two were not able to be fully realized, and this may have contributed to the lack of greater improvement in reporting accuracy between phases one and two. Research has shown that satisfaction with training is indicative of community health worker capacity and motivation, so the RMM training process would need to be improved if it is scaled up [26,27].

The data management process also required continuous assessment and support. For this reason, phase two included data management changes to improve data quality and the efficiency of the data management process. Despite these reinforcements, results did not fall within the acceptable range. Communication between the NSO and HSAs was also strengthened early on in the project, when the data editor was given the role and materials to communicate with HSAs by phone in order to notify them of field visits and to follow up on data quality issues. This improved the timeliness with which data quality issues were resolved, but cell phone network limitations and limited access to monthly airtime vouchers affected the data editor’s ability to follow up and confirm some events in a timely fashion.

The advantage of building RMM within an existing government structure also resulted in some important challenges. The integrated design hampered the efficiency of the data management process, because the shortcomings of the existing system affected RMM project operations. We worked primarily with Ministry of Health employees at the district level. As a result, there was limited control over these staff members, whose first responsibility was to their government duties. The scheduling of review meetings, submission of extraction forms, and completion of supervision were sometimes delayed or cancelled due to HSA and supervisor task shifting when activities such as indoor residual spraying or in-service training took priority over RMM. Surveillance of vital events is best conducted without interruptions, which cannot occur when HSAs are required to temporarily modify their work routine.

RMM also faced frequent staff turnover at all levels of the project and had little control over these personnel changes. The local NSO principal investigator for the project and district coordinators changed multiple times, with negative effects on coordination, management, and communication. At the community level, 28% of catchment areas experienced at least one turnover of the HSA during the project period. Research has shown that program continuity is hampered by turnover [27].

Full implementation of community-based reporting of vital events would not mean that other methods of collecting mortality data could be abandoned. High-quality survey data with sufficient power to validate results would be needed to evaluate the completeness of estimates. The RMM data management team at the NSO found it challenging to manage both the continuous RMM data management tasks and simultaneously manage the midline and endline surveys, which may have affected data quality of the latter.

The comparison of survey results for the first four annual periods suggests that endline validation survey interviewers did not capture all events. Also, the endline survey estimate of U5MR of 60 per 1,000 live births in 2013 suggests a dramatic halving of child mortality over the last few years in Balaka. Such a notable decline seems unlikely. This suggests that the ratio results comparing the community-based RMM method and the endline validation survey are overestimated, particularly for the later annual periods, and an adjustment of ratios would result in even more modest validation results for the HSA community-based method. We did not conduct a record linkage exercise between the HSA vital event reports and the validation surveys, so it is not possible to quantify the magnitude, or describe the characteristics of, events that were missed by the HSA and the current best practice surveys. We attempted to use results from the Millennium Development Goal survey conducted from November 2013 to April 2014 by the NSO with support from UNICEF and other partners, but the sample sizes of the survey did not provide sufficient power for it to be used to validate the phase two results.

The operational realities that resulted in poor data quality pose important sustainability challenges to community-based health workers and data management staff at the NSO. Further, pregnancy histories are not easy to implement as part of a validation survey and can be hampered by substantial non-sampling errors, as was the case in Balaka. However, even if the pregnancy histories underestimated annualized U5MRs in 2013, our results still show that the vital events data collected by HSAs consistently and significantly underestimated annualized U5MRs.

## Conclusions

The RMM experience in Balaka and Salima districts demonstrates that HSAs have the potential to generate data to support estimates of under-five mortality to provide timely data to policy makers while the recently enacted vital event registration system is being fully implemented. However, our findings indicate that the current system needs substantial strengthening to ensure that birth and death reporting by HSAs is sufficiently complete over time to ensure reasonably accurate estimates of annualized U5MR. The optimum level of incentives and support to make the system work while ensuring sustainability is extremely difficult to find. We invested additional resources in phase two to identify and implement these supports and incentives but did not find the optimum level. Policy makers and program managers in Malawi can use lessons learned from RMM implementation to improve the HSA system and supports for improved outcomes.

## Supporting Information

S1 File Situation analysis. (DOCX)

S2 File RMM data management process and supervision. (DOCX)

S3 File Village Health Register Verification surveys. (DOCX)

S4 File Validation surveys. (DOCX)
