# Supplementary material for: Strengthening Community-Based Vital Events Reporting for Real-Time Monitoring of Under-Five Mortality: Lessons Learned from the Balaka and Salima Districts in Malawi
Source: PLoS One. 2016 Jan 11;11(1):e0138406. doi: 10.1371/journal.pone.0138406 (PMC4713469; doi:10.1371/journal.pone.0138406)
Supplement: S1 File — (DOCX) [file pone.0138406.s001.docx]

**S1: Situation analysis**

An external consultant conducted a situation analysis after results from the midline validation showed under-reporting of under-five mortality by HSAs participating in RMM. The consultant conducted a document review and interviews with HSAs and stakeholders to identify the six limitations to be addressed for improvements in RMM.

1. HSA ownership of RMM is weak and can be strengthened by improving community level support and reviewing key RMM messages with HSAs. The NSO should present RMM to district authorities and the Village Health Committees at the community level so that HSAs receive community level support.
2. Supervisors are not completing their defined tasks and would benefit from guidance and feedback.
3. HSAs are not using the Village Health Register (VHR) properly and need new materials and re-training on the data management process.
4. The HSA’s relationship with his/her community is important and should be strengthened by training the Village Health Committee on RMM.
5. HSA data quality standards have weakened since the start of RMM. Data quality feedback should be provided at the district level, when presenting results at the review meetings, and at the individual level through report cards.
6. HSAs are recognized as important community-based health agents and face task shifting challenges due to this role.

These limitations were presented along with midline validation results at a stakeholder’s meeting during which it was agreed that RMM has the potential to capture complete under-five mortality but needs improvements. The following improvements were developed and implemented for phase two which was introduced at the September 2012 review meeting. Improvements were rolled out through July 2013.
