# Supplementary material for: Strengthening Community-Based Vital Events Reporting for Real-Time Monitoring of Under-Five Mortality: Lessons Learned from the Balaka and Salima Districts in Malawi
Source: PLoS One. 2016 Jan 11;11(1):e0138406. doi: 10.1371/journal.pone.0138406 (PMC4713469; doi:10.1371/journal.pone.0138406)

**S2: RMM data management process and supervision**

**Data collection*.*** HSAs are expected to identify pregnancies, births, and deaths within their catchment areas and record the information in their VHR (Figure 1). The HSAs extract the information from the registers every month using a RMM extraction form (Table S1). To familiarize themselves with their community, HSAs began their RMM activities by listing members of each household within their area in the VHR. Each household and family member was assigned a code, represented by a sequence of 11 digits with the zone (1 digit), the district (1 digit), the traditional authority (2 digits), the Group Village Head (2 digits), the household (3 digits), and the household member (2 digits). HSAs submitted the completed extraction form to the responsible supervisor, who checked the data and submitted the form to the district RMM coordinator. In RMM phase one, the district coordinator made a photocopy of the extraction forms and sent the original forms to the NSO. In RMM phase two, HSAs used carbon copy extraction form booklets that contained two copies in addition to the original. The original was sent to the NSO, one copy was kept by the district coordinator, and the one copy was kept by the HSA.

The NSO collected the RMM extraction forms every month from the district, and maintained a spreadsheet to monitor the completeness of reporting (Figure S2). After the spreadsheet was updated, the data editor at the NSO reviewed each extraction form for completeness and errors. He then called HSAs with errors noted on their extraction forms, or those who documented adverse events such as a neonatal death, stillbirth, abortion, miscarriage, and maternal death. Once the HSA had clarified and/or confirmed the event, the data editor submitted the checked forms for data entry in CSPro. To minimize data entry errors, double independent data-entry was performed and any differences reconciled through a review of the original data.

The Principal Investigator at NSO cleaned the reconciled data with the support of the data quality team at IIP. A data quality assessment was conducted for all records, and potential errors, omissions, and inconsistencies were recorded and sent to the data editor. The data editor resolved these issues through a review of the extraction form or a phone call to the relevant HSA, and responded to each issue identified through the data quality assessment. These resolutions were submitted in writing to the Principal Investigator, who edited the data and performed a final check to produce a clean dataset.

**Supervision***.* The district RMM coordinator assigned each HSA working on RMM to a specific supervisor at the health center. The supervisor checked HSA performance, how well he/she completed the extraction forms, whether births and deaths were recorded in the VHRs, made corrections if needed, and provided feedback and on the job training in RMM procedures.

The NSO team visited the districts or the HSAs monthly to collect and provide new extraction forms, and to follow up on transfers of HSAs and if so, to check whether they had been replaced.

RMM data review meetings were held regularly throughout the implementation period in both districts. All HSAs were invited, along with Ministry of Health officials, the District Health Officer, the HMIS officer, and other partners such UNICEF and WHO. During the review meetings, reports were made on progress in HSA reporting of pregnancies, births and deaths, and feedback was provided. HSAs and Supervisors were given the opportunity to discuss challenges, issues, and suggested solutions. The NSO used the data review meetings as an opportunity to provide refresher training on RMM procedures and the data flow process and to reinforce the importance of ensuring data quality in terms of accuracy, reliability, and completeness. During RMM phase two, data review meetings were scheduled for once a quarter. At the September, 2012 and subsequent quarterly data review meetings, all activities remained the same as in phase one, but phase two materials were distributed, incentives were presented, and trainings for phase two activities were held. Some phase two activities such as the Village Health Committee training and the Supervisor Checklist had follow-up trainings that the NSO data management team conducted during the monthly field visits to collect extraction forms.

**Figure S1. Pages from Village Health Register**

**
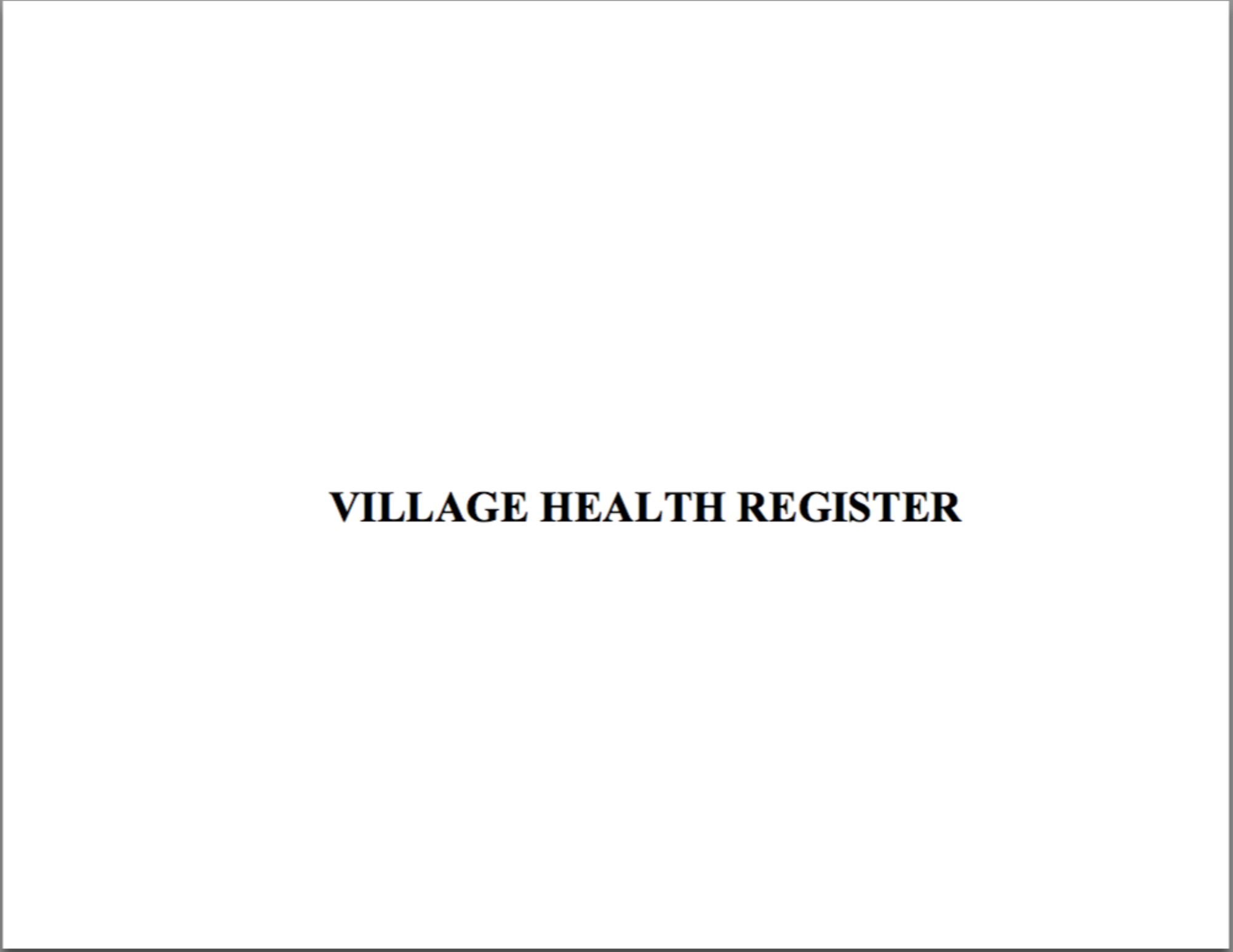
**

**
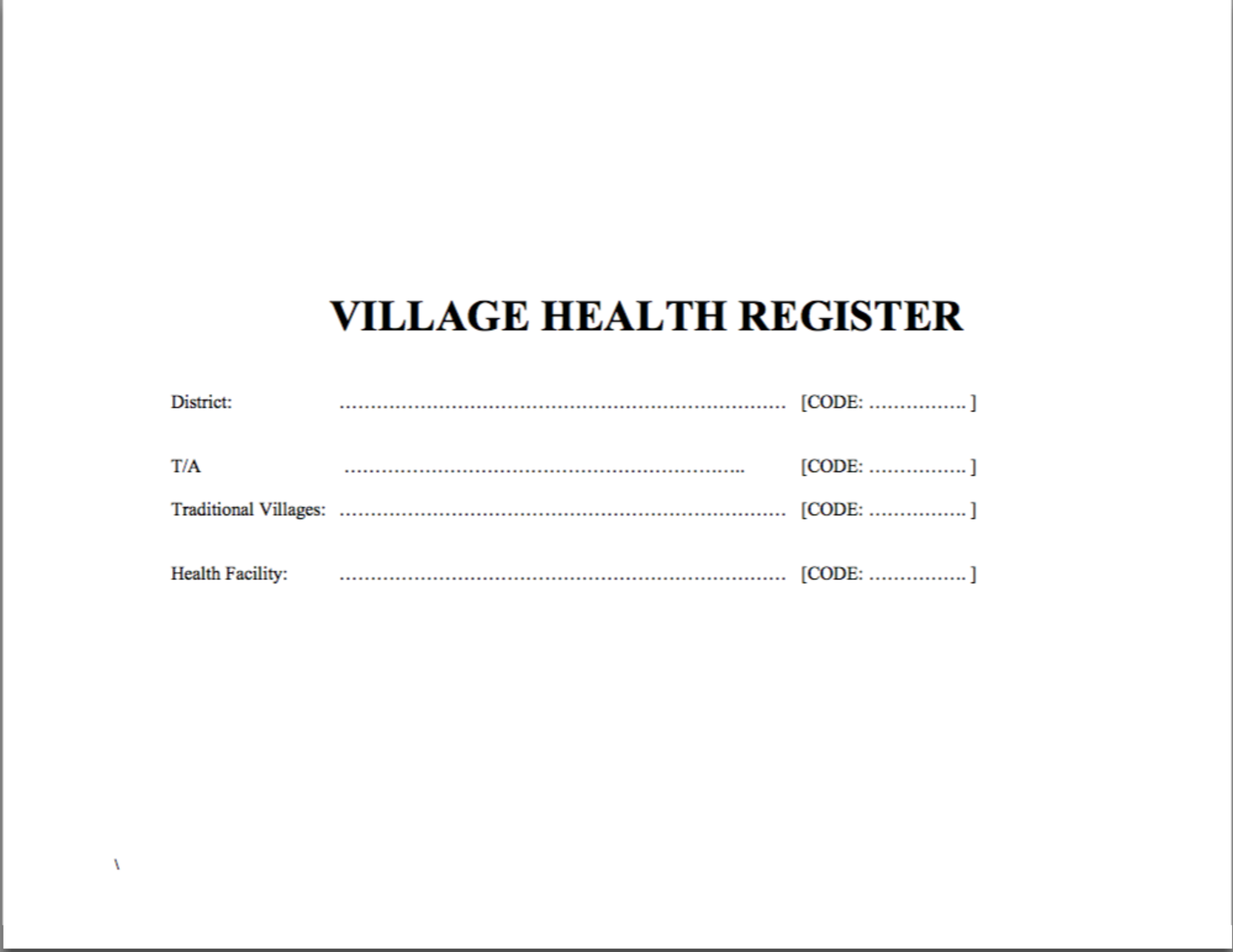
**

**
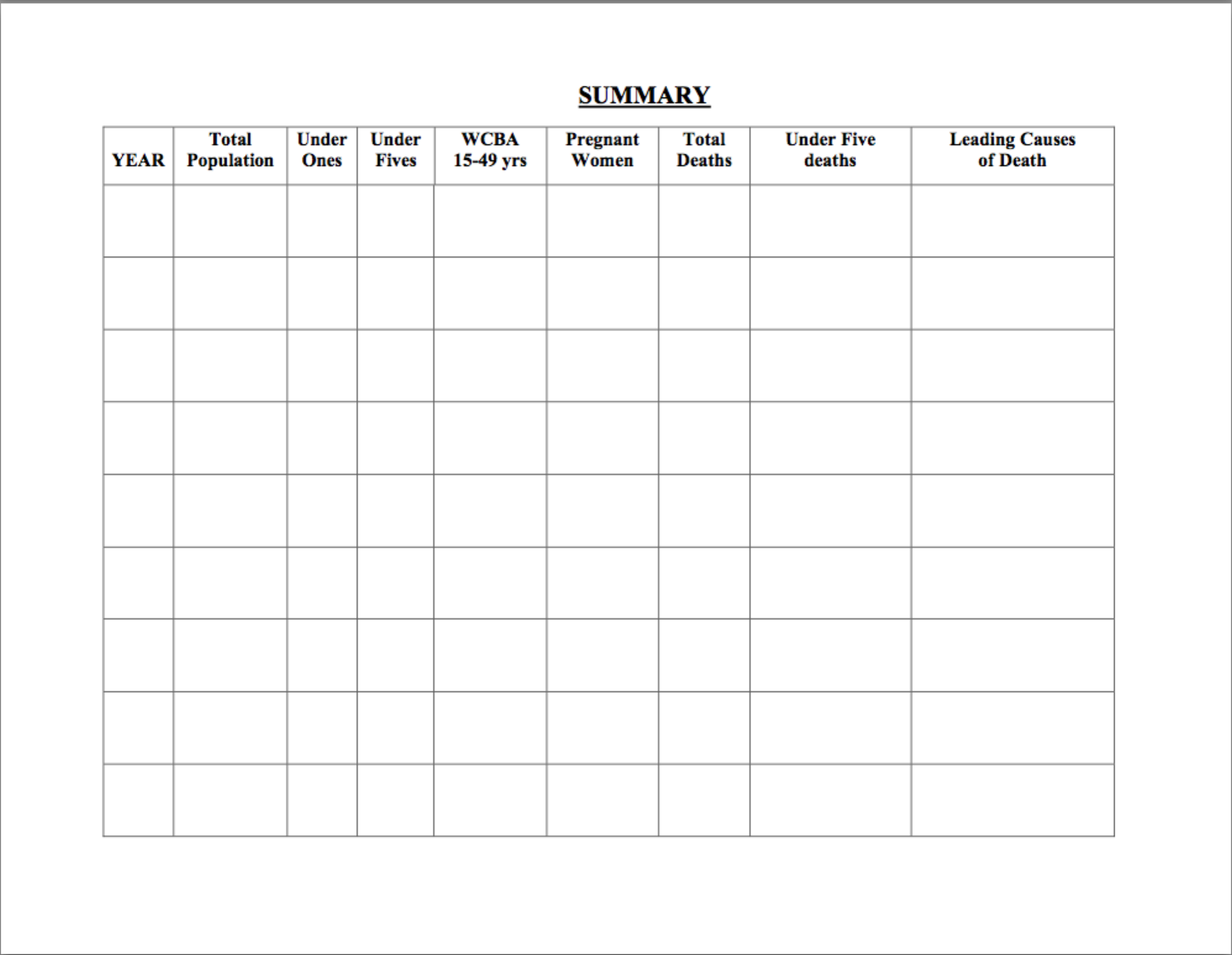
**

**
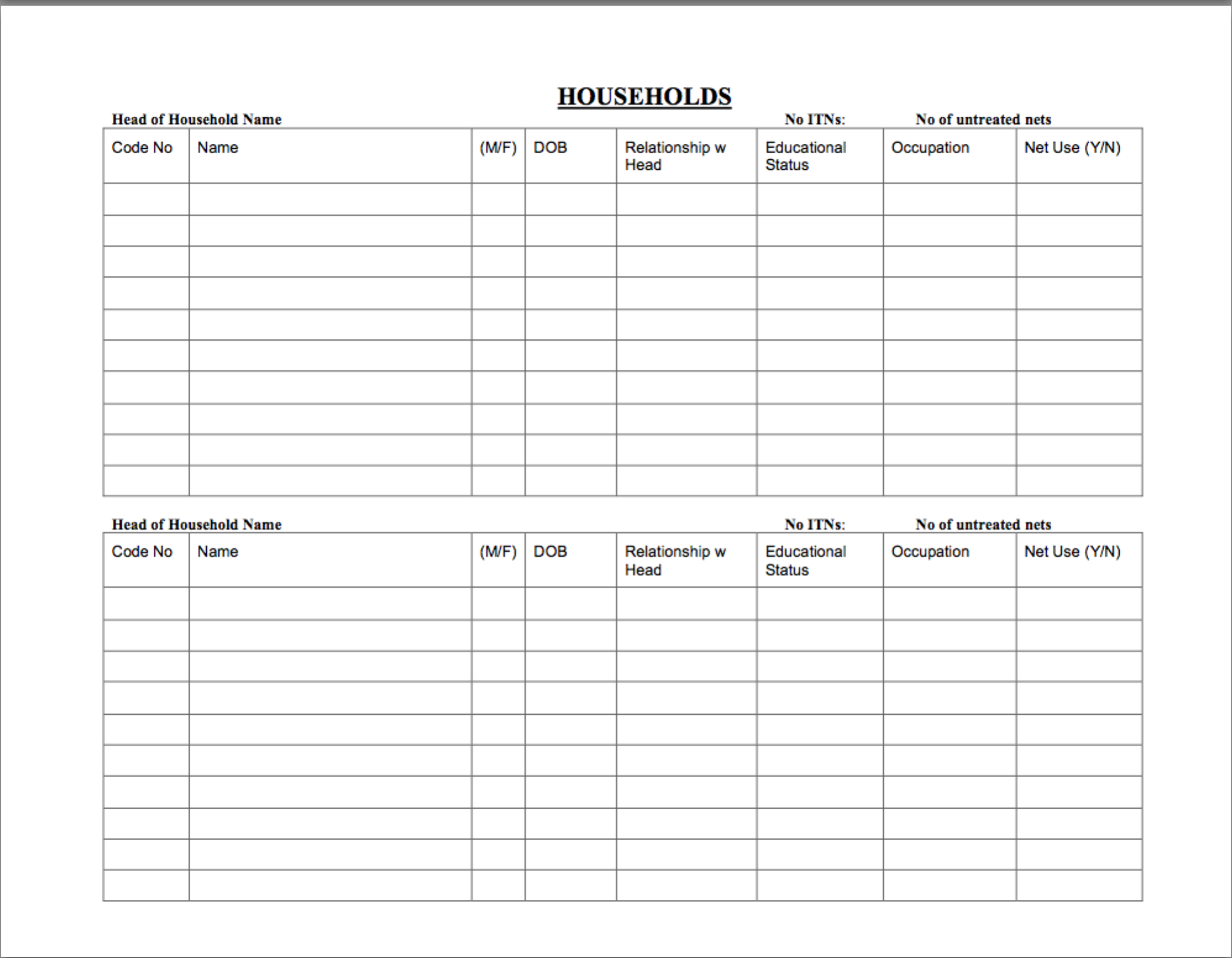
**

**
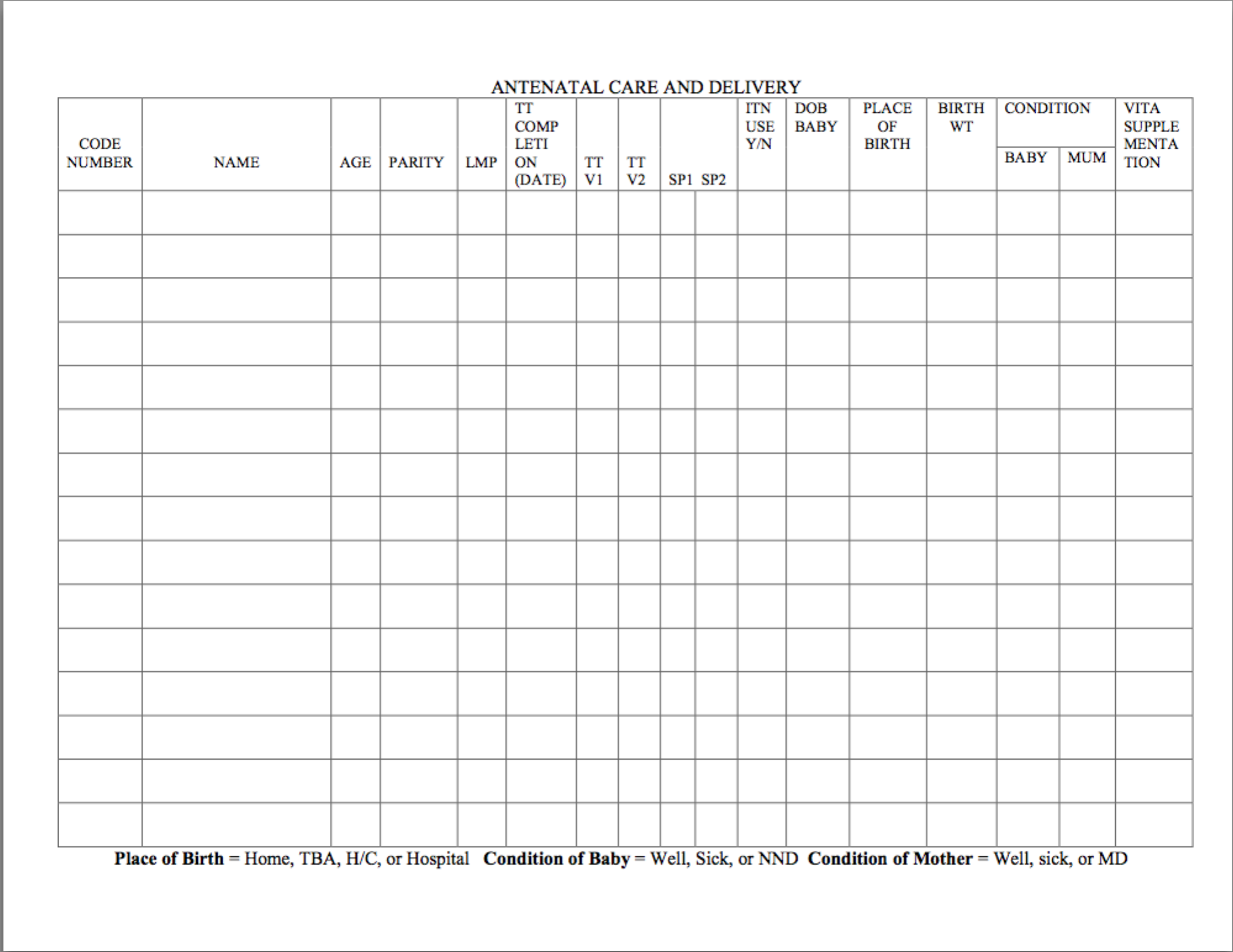
**

**
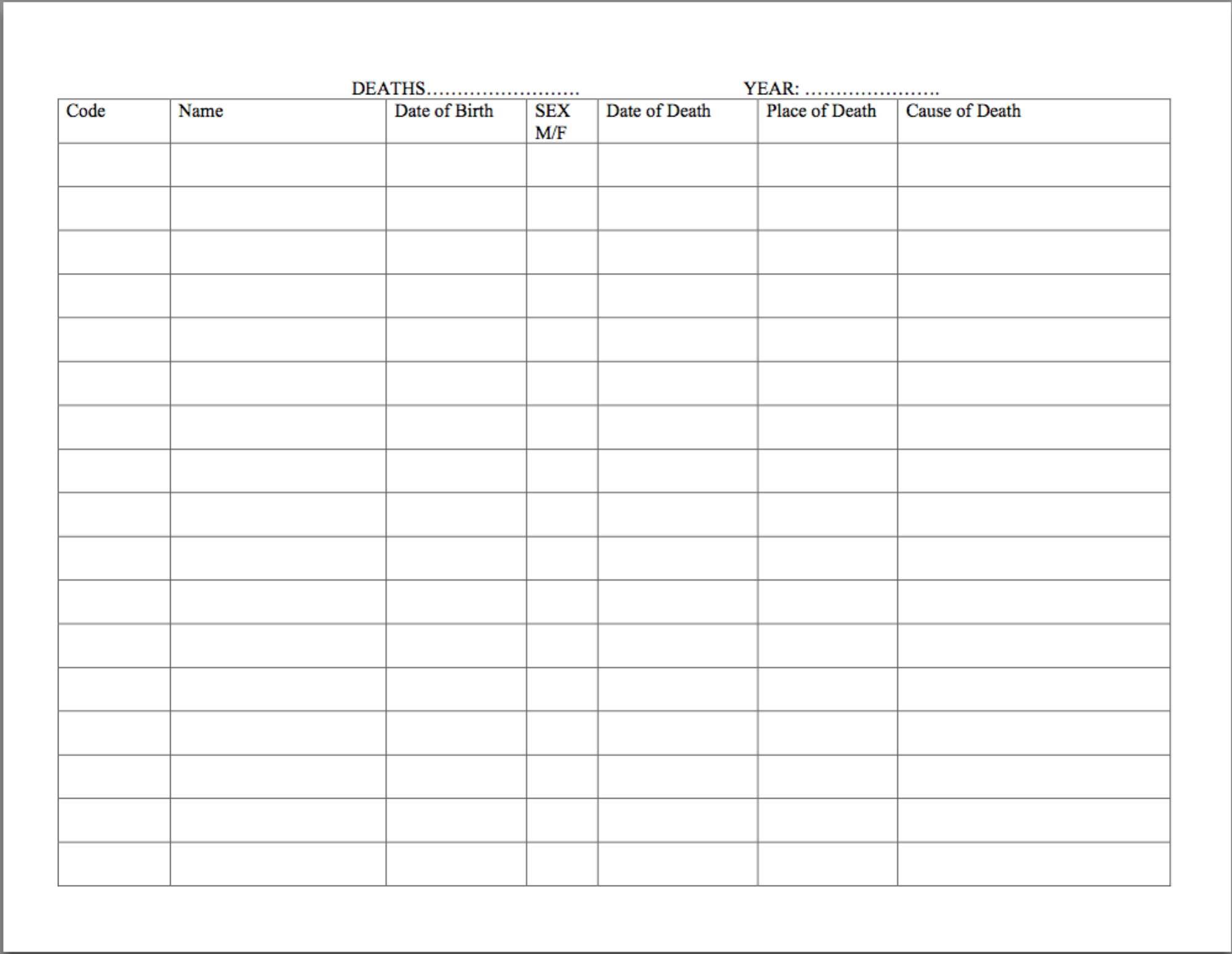
**

**Table S1. RMM Extraction Form**
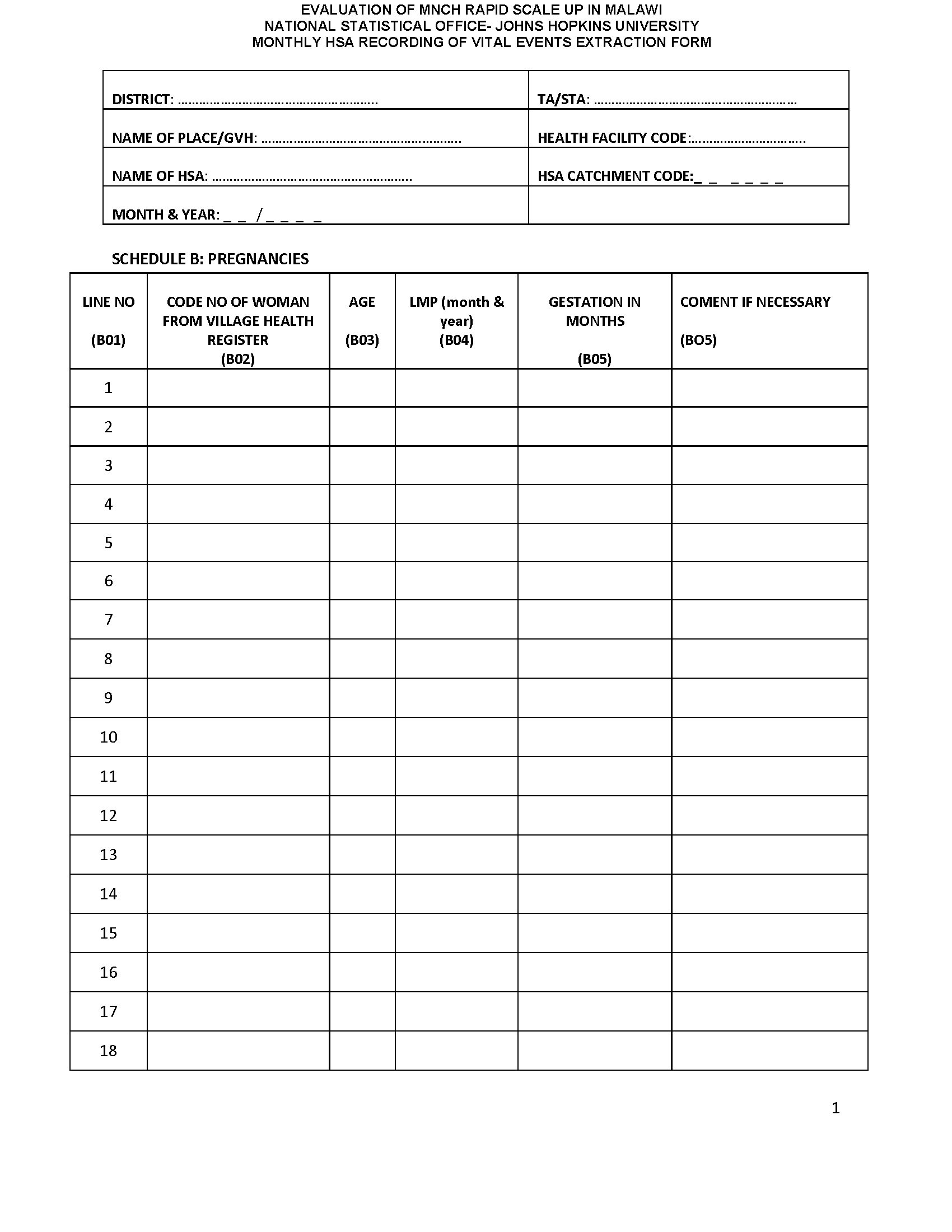


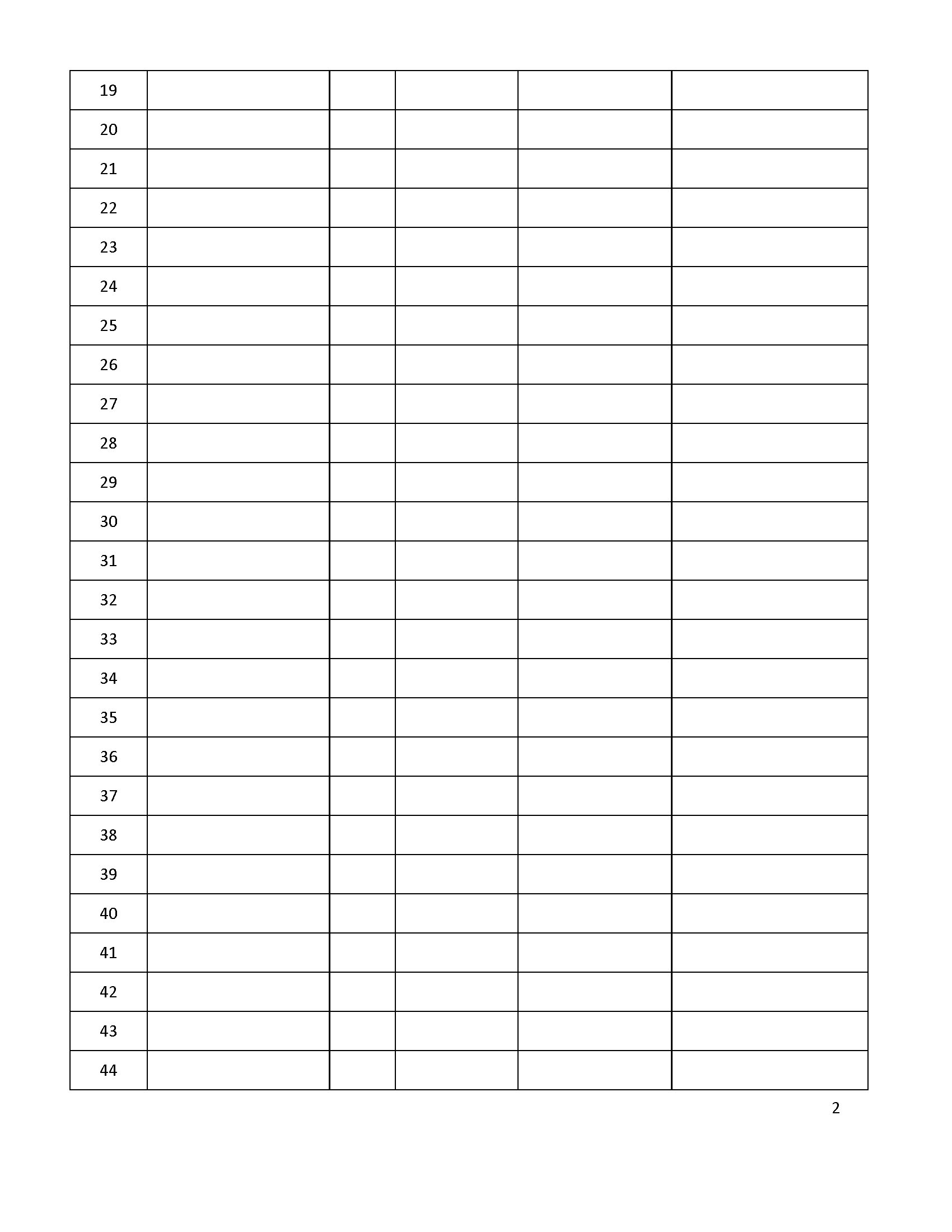

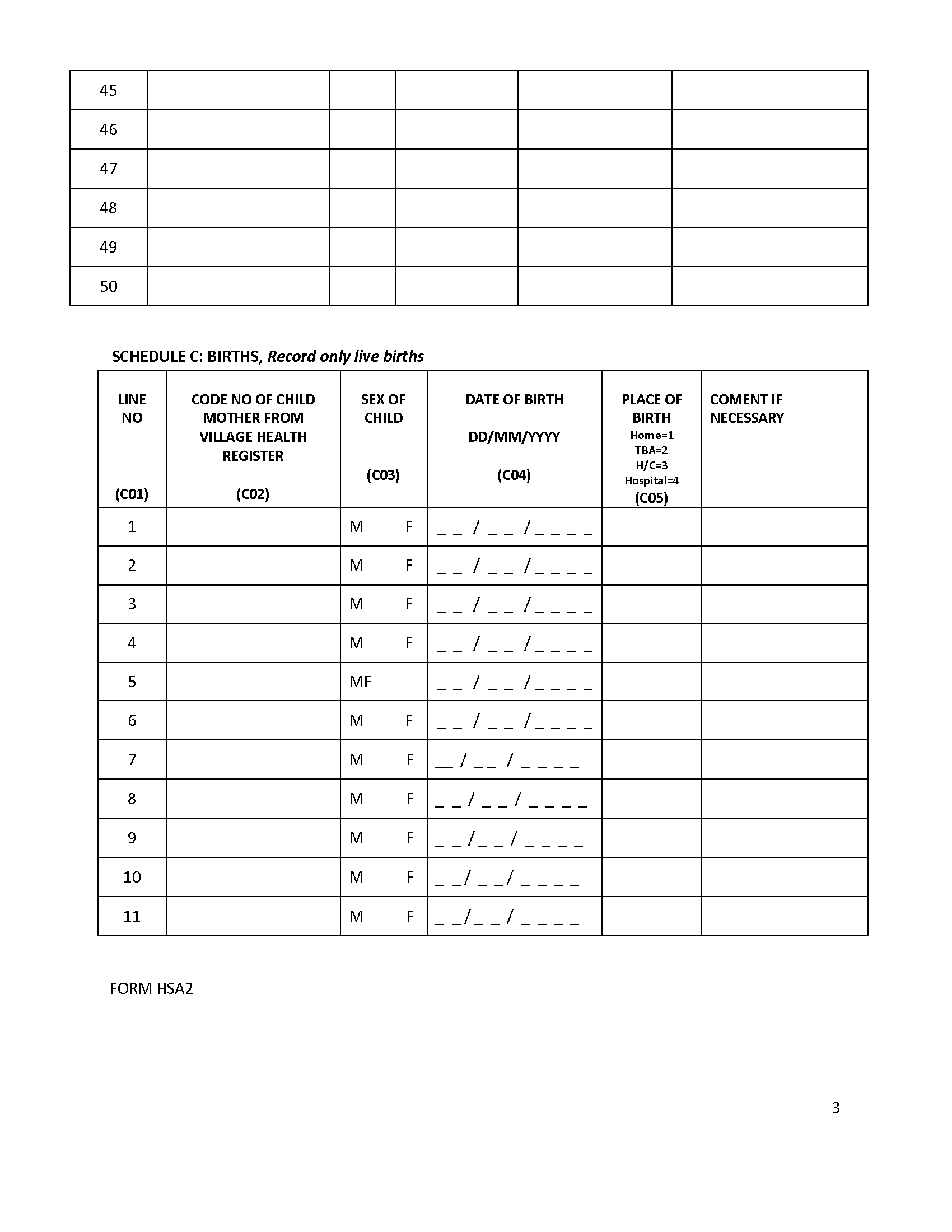

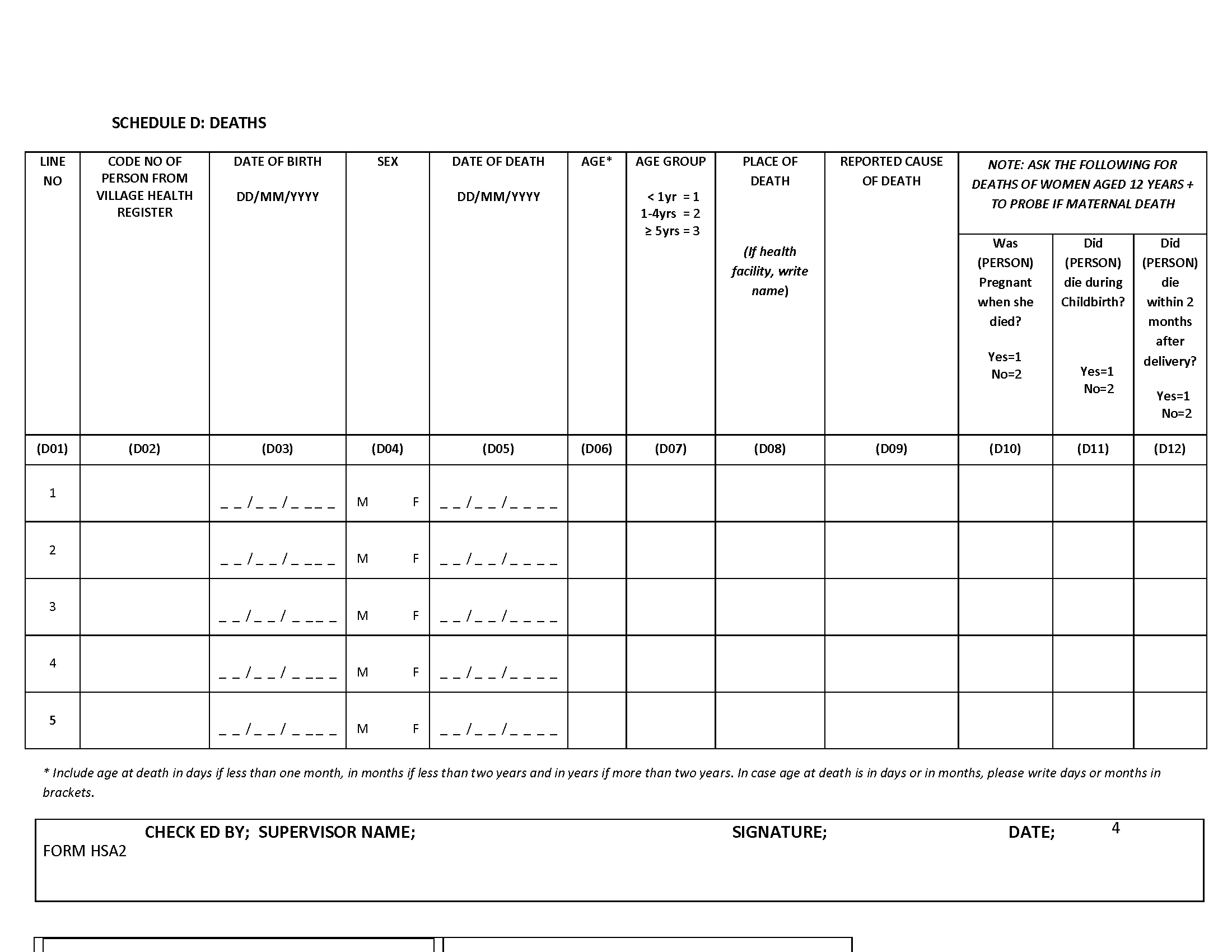


**Figure S2. Extraction form submission.** Number of HSAs submitting extraction forms to the NSO by month an district from January 2010 through December 2013


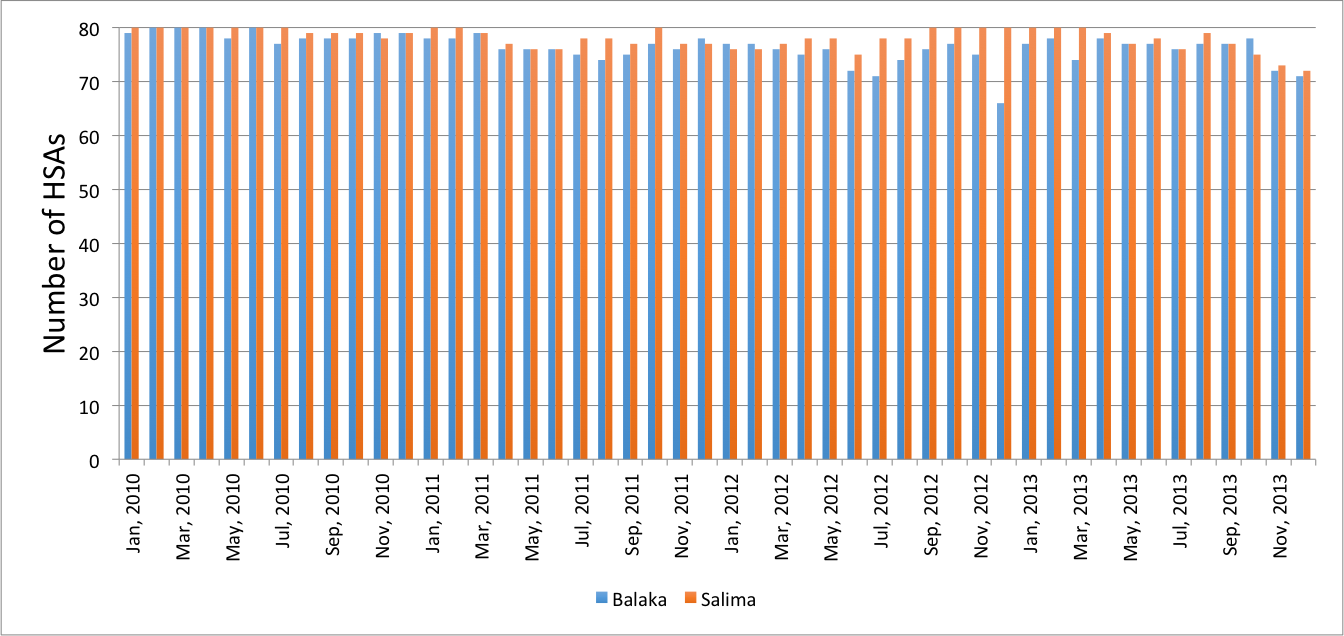

Supplement: S2 File — (DOCX) [file pone.0138406.s002.docx]
