# Supplementary material for: Strengthening Community-Based Vital Events Reporting for Real-Time Monitoring of Under-Five Mortality: Lessons Learned from the Balaka and Salima Districts in Malawi
Source: PLoS One. 2016 Jan 11;11(1):e0138406. doi: 10.1371/journal.pone.0138406 (PMC4713469; doi:10.1371/journal.pone.0138406)
Supplement: S3 File — (DOCX) [file pone.0138406.s003.docx]

**S3.** **Village Health Register Verification surveys**

Two field-based assessments of HSA data recording practices, referred to as the VHR Verification, were conducted and also included a questionnaire on HSA and community characteristics. The phase one assessment was conducted after 24 months of implementation of the community-based RMM method, from January 10^th^, 2012 to January 19^th^, 2012. The phase two assessment was conducted in July, 2013. The aim of the assessments was to determine whether HSAs accurately reported on, and correctly documented, vital events in their VHR and the monthly extraction forms. The specific objectives of the VHR Verifications were:

- To document consistency between records of events in the VHR and the data reported by HSAs to NSO on monthly extraction forms; and
- To verify the accuracy of vital events reported to NSO.

**Methods.** Prior to fieldwork, the NSO data management team prepared a spreadsheet of vital events data submitted by HSAs to the NSO, to be used in checking the HSAs’ VHRs, and during household accuracy checks. The spreadsheet included births reported by each HSA from the last three extraction forms submitted to the NSO. Due to some extraction form submission delays, the last three forms were not necessarily from the most recent three months. Additionally, all under-five deaths reported by each HSA in the past 12 months were included in the assessment spreadsheet.

The NSO team visited each HSA and checked each of the births and deaths on the assessment spreadsheet against data recorded in the VHR. Once the VHR Verification was completed, the NSO team conducted household accuracy checks with the HSA. Together they visited the homes where three randomly chosen births and under-five deaths occurred, to confirm the events and the accuracy of the dates when the events were reported to have occurred. For HSAs reporting less than three births during the eligible period, less than three births were followed up at the household. For HSAs reporting three births or less, the births followed up at the household were not randomly selected. The identification of the home address of each selected event was based on the household’s code included in the VHR, complemented by the HSA’s knowledge of the community. Prior to the field visit, the NSO team informed district officials (e.g., the DHO and RMM coordinator) about the upcoming exercise.

**Results.** The response rate for both VHR Verification assessments was high, though participation improved 12.5 percentage points between phase one and phase two (Table S2). Among the interviewed HSAs, only about half lived in the catchment area they served. Catchment area residence is one of the HSA requirements set by the MOH.

**Table S2. Response rate and catchment area residence**

|  | **District** | | | | **Percentage Point Difference** |
| --- | --- | --- | --- | --- | --- |
| **Variable** | **Phase I** | | **Phase II** | |  |
|  | n | % | n | % |  |
| Response Rate for HSA questionnaire | 160 | 81.3% | 160 | 93.8% | 12.5% |
| Proportion of HSAs who live in catchment area | 130 | 54.0% | 150 | 46.7% | -7.3% |

For each assessment, the NSO data management team checked just over 1,000 births reported to the NSO in the HSA VHRs. In VHR Verification one, 1016 births were checked (Table S3). In phase two, 1211 births were checked. Most of these births reported to the NSO were found in the HSA VHRs; 92.3% in phase one and 83.5% in phase two. Among these audited births, most had a date of birth in the VHR consistent with the date of birth reported to the NSO. Once the NSO interviewer finished checking births and date of birth information in the VHR, the interviewer went with the HSA to the household of the randomly selected birth to confirm the event and the date of birth. Almost all births were confirmed at the household, Dates of birth reported by families were consistent with most of the dates of births reported to the NSO by HSAs.

In phase one, 69.2% of HSAs had all the births checked in the VHR Verification assessment also documented in the VHR. Poor documentation practices are therefore not limited to a few HSAs but, instead, spread across about 30% of the interviewed HSAs. Phase two had similar results, though improvements were found in the documentation of date of birth. Almost 50% of interviewed HSAs had inconsistent date of birth information reported to the NSO that did not match with date of birth information in the VHR. In phase two, only about 30% of HSAs had inconsistencies between documented and reported dates of birth.

**Table S3: Birth verification results**

|  | **District** | | | | **Percentage Point Difference** |
| --- | --- | --- | --- | --- | --- |
| **Variable** | **Phase I** | | **Phase II** | |  |
|  | n | % | n | % |  |
| Proportion of births in NSO records included in the VHR | 1016 | 92.3% | 1211 | 83.5% | -8.8% |
| Proportion of audited births for which the date of birth was consistent with NSO records | 1016 | 85.9% | 1211 | 89.8% | 3.9% |
| Proportion of audited births confirmed in the HSA catchment area | 335 | 100.0% | 412 | 97.8% | -2.2% |
| Proportion of audited births confirmed in the HSA catchment area with consistent date of birth | 335 | 94.9% | 385 | 93.9% | -1.0% |
| Proportion of HSAs with all audited births included in VHR | 130 | 69.2% | 144 | 67.4% | -1.8% |
| Proportion of HSAs with date of birth of all audited births consistent with NSO records | 130 | 48.5% | 144 | 72.9% | 24.4% |

Though methods for under-five mortality audit selection were similar for assessments in phases one and two, phase two had about half the number (n=223) of under-five deaths identified for auditing in the assessment (Table S4). Most of the deaths in NSO records were found in VHRs, 90.5% in phase one and 87% in phase two. Once the NSO interviewer finished checking under-five deaths in the VHR, the interviewer went with the HSA to the household of the randomly selected under-five death to confirm the event. Almost all under-five deaths were confirmed at the household, though results were not as high in phase two.

In Phase I, 75.5% of HSAs had all of the under-five deaths checked in the VHR Verification assessment also documented in the VHR. Poor documentation practices are therefore not limited to a few HSAs but, instead, spread across about 25% of the interviewed HSAs. There was 6.3 percentage point improvement between assessments in phases one and two in the proportion of HSAs that had all audited deaths included in VHR.

**Table S4. Death verification results**

|  | **District** | | | | **Percentage Point Difference** |
| --- | --- | --- | --- | --- | --- |
| **Variable** | **Phase I** | | **Phase II** | |  |
|  | n | % | n | % |  |
| Proportion of deaths in NSO records included in the VHR | 423 | 90.5% | 223 | 87.0% | -3.5% |
| Proportion of audited deaths confirmed in the HSA catchment area | 222 | 98.6% | 194 | 94.8% | -3.8% |
| Proportion of HSAs with all audited deaths included in VHR | 102 | 75.5% | 99 | 81.8% | 6.3% |

**Conclusions.** The results of assessments in phases one and two demonstrated that HSAs correctly documented births and deaths most of the time. The phase one results were incorporated into the midline validation result findings, which were used to inform program improvements for phase two. The phase two assessment was conducted prior to the end of RMM to assess improvements since VHR Verification one. Few improvements were noted. In fact, most of the assessment indicator results in phase two were worse than in phase one. Further research is needed to identify the determinants of data quality for the selection of appropriate incentives and support.
