# Supplementary material for: Strengthening Community-Based Vital Events Reporting for Real-Time Monitoring of Under-Five Mortality: Lessons Learned from the Balaka and Salima Districts in Malawi
Source: PLoS One. 2016 Jan 11;11(1):e0138406. doi: 10.1371/journal.pone.0138406 (PMC4713469; doi:10.1371/journal.pone.0138406)
Supplement: S4 File — (DOCX) [file pone.0138406.s004.docx]

**S4. Validation surveys**

### Midline validation survey

The main objective of the midline household mortality survey was to obtain the data needed to validate the three RMM approaches implemented in two districts in Malawi. More specifically the survey was designed to:

- Estimate childhood mortality in the districts of Balaka and Salima, based on women’s report of their full birth history; and
- Support validation of the RMM methods implemented in Balaka and Salima.

**Sample size.** We assessed the validity of the routine data recorded by HSAs by comparing the annual under-five mortality rate computed based on data from village health registers to the under-five mortality rate computed from the household survey. A test of equivalence between the mortality rate generated from the HSA data (p1) and the rate produced by the household survey (p2) was used to estimate the sample size required to determine whether p1 is within 20% of p2, the acceptable margin of equivalence. More precisely, we assumed that the two methods were equivalent if p1-p2 fell within a set confidence interval of (-0.2xp2; 0.2xp2). Assuming an under-five mortality rate of 0.120, this implies that p1-p2 must fall within a 95% confidence interval of (-0.024; 0.024) with 80% power to reject the hypothesis of non-equivalence between the two methods. SAS was used to compute the corresponding sample size using simulations with 100,000 replications. Table S5 indicates that a sample size of 30,000 households corresponding to 160 HSAs (increased by 7% to account for transfers and loss to follow up) was required for the test of the RMM method based on village health registers. For the current best practice household survey, a sample size of 24,000 households was required in both districts.

**Table S5. Sample size calculations**

| Parameters | HSA Recording method | Midline survey | Endline Survey |
| --- | --- | --- | --- |
| Mortality rate | 0.120 | 0.120 | 0.100 |
| Difference to detect | 0.2 | 0.2 | 0.2 |
| Confidence level | 0.95 | 0.95 | 0.95 |
| Power | 0.8 | 0.8 | 0.8 |
| Cluster size (births) | 40 | 21 |  |
| Design effect | 2 | 1.5 | 1.5 |
| Crude birth rate | 0.04 | 0.04 | 0.04 |
| Average household size | 5 | 5 | 5 |
| Non-response rate | 0 | 0.05 | 0.1 |
|  |  |  |  |
| Number of births | 6,000 | 5000 | 2000 |
| Corresponding number of households | 30,000 | 24,000 | 10,000 |
|  |  |  |  |
| Corresponding number of HSA, increased by 7% to account for transfers and loss to follow up | 160 |  |  |

**Sampling frame and sample selection.** The 2008 Population Census frame was used to select the primary sampling units or enumeration areas (EA) for the survey, with probability proportionate to size. The sampling was stratified by district.

The plan was to select 35 households per EA, making a total of 686 EAs, equally divided across the two districts. Thus, 343 EAs were selected in each district. In Balaka, the total number of EAs in the entire district was 294. However, there were large variations in the size of EAs, ranging from 21 to 650 households with an average number of 253 households. To reach the 343 EAs needed in the sample, large EAs with more than 330 households were divided into two or three EAs, and all resulting EAs in the district were included in the sample (now a census of EAs). All households within each EA were listed and a sample of 35 households was selected using a systematic random sampling procedure. Thus, in Balaka, only a single stage sample of households was conducted.

In Salima, the total number of EAs is 435. Two-stage sampling was used, with EA selection at first stage and household selection at a second stage. The primary stage selection was conducted using a systematic random sampling procedure with probability proportionate to size in terms of households. The size of EAs varied from 15 to 780 households. During the sample selection, some very large EAs were selected twice; one EA was selected three times and another four times. These EAs were divided into the number of times they were selected, and each segment used a separate cluster. In addition, very small EAs were combined with neighboring EAs before the random selection was completed. All households in each selected EAs were listed and a random selection of 35 households occurred using systematic random sampling procedures.

Given that the same number of households was selected in each cluster, the probability of selection of a household was not the same for every home included in the sample. Thus, sampling weights were computed and used during analysis to ensure the representativeness of the results.

**Questionnaires.** A structured, pre-coded questionnaire adapted from the standard DHS tool was used. The household form collected basic information about each household member as well as information about access to water and sanitation facilities, roof, floor, and wall construction materials, number of sleeping rooms, possession of land, livestock ownership, and household ownership of assets such as mobile phones, bicycles, radios, and others items. GPS coordinates were collected for each household.

The “individual woman” form was administered to all women ages 15-49 living within the household. Women were asked to provide their age, marital status, and educational level. They were asked a summary list of questions on whether they had ever given birth, and if so, how many of their children were still alive at the time of the interview. If a woman reported having children, a full birth history module was administered that included detailed questions about each child.

All questions were written in both English and the local language of Chichewa to assist the interviewers, but most interviews were conducted in Chichewa. The tool was pre-tested during interviewer field practice sessions in rural villages near Zomba in October, 2011. To ensure the highest possible survey quality, the questionnaire was kept short, and administration took 15 to 30 minutes.

### Endline validation survey

To reassess the accuracy and reliability of data collected in measuring child mortality, an endline validation survey was conducted using a pregnancy history in all of the RMM HSAs’ catchment areas. This survey was conducted from November, 2013 to January, 2013 in both districts to validate under-five mortality rates from HSA records. The objectives of the endline validation survey were to:

- Assess the level of completeness of reporting on pregnancies, births, and deaths recorded by HSAs in their VHRs and reported to NSO;
- Determine the patterns of births and deaths that are likely be missed by HSAs;
- Compare neonatal and under-five mortality rates from data reported by HSAs and the endline survey; and
- Assess the relationship between HSA and catchment area characteristics and the quality of data reported on pregnancies, births and deaths.

**Sample size.** A sample size of 10,000 women was needed for the pregnancy history and was divided equally by district (Table S5). Within each district, the number of households selected in each of the 160 HSA catchment areas was proportional to the total household size of the catchment areas. In addition to the pregnancy history sample size of 10,000 women aged 15-49, the endline survey also included a household survey with a sample size of 10,000 subjects. In total, 9,929 subjects participated in the household interview, 9,659 women aged 15-49 participated in the women’s interview, and 150 HSAs participated in the HSA interviews. The response rate superseded the minimum requirement of 9,000 participants for the household and women’s interviews.

**Sampling frame and sample selection.** The endline household survey used a stratified two-stage cluster sampling design with the HSAs’ catchment areas serving as clusters. The primary sampling units (PSU) were the HSA catchment areas, which were selected with a probability of 1.0. The secondary sampling units (SSU) were randomly selected households within the PSUs. The sample size of the SSUs was calculated using probability proportional to the number of households in the catchment area.

According to guidelines set by the Malawian Ministry of Health (MOH), HSAs are responsible for a population of 1000 inhabitants, which is roughly equivalent to 200 households. However, HSAs reported their catchment area population on a regular basis, and their estimates average around 1200 inhabitants per catchment area. IIP-JHU used HSA catchment area population estimates from July, 2012 to estimate the SSU sample size for each PSU. The NSO Central Team provided this information to the supervisor of each interviewer team, because the random selection of households was conducted in the field. Once the enumerators completed the HSA catchment area household listing, the supervisor used the SSU sample size provided by the NSO Central Team and a password protected Excel spreadsheet to select randomly the households to be surveyed in the catchment area. Within each household, the head of household (or a comparable adult) was interviewed using the Household Questionnaire, and women aged 15-49 were interviewed using the Women’s Questionnaire which included a full pregnancy history.

**HSA questionnaire.** All RMM HSAs were interviewed to capture HSA characteristics, supervision, RMM support, and perceptions of community engagement and communication with the HSAs. The sample for the HSA interviews was a census of the HSAs responsible for the 160 catchment areas randomly selected for RMM. Some catchment areas did not have an HSA at the time of the survey, so survey teams in those catchment areas did not conduct the HSA interview. The intended sample size was 160 HSAs; response rates were an average of 94% in both districts due to catchment areas with unavailable HSAs and those without an actively serving HSA. The HSA questionnaire was developed by an IIP-JHU team member and reviewed by NSO RMM data management team members. This questionnaire covered topics including HSA characteristics, community characteristics, supervision and RMM support, vital event documentation practices, and HSA perceptions of RMM phase two activities and community engagement with HSAs. The NSO RMM officer and IIP technical team member pre-tested the questionnaire in the field among three HSAs in the Balaka district, and modified it based on the results. The final questionnaire was in English because HSAs are proficient in English and it is the language used for extraction forms. The endline survey team supervisor or technical resource person administered the questionnaire when the HSA was available during the interviewer team’s stay in the HSA’s catchment area.
